# Analysis on the Interaction Domain of VirG and Apyrase by Pull-Down Assay

**DOI:** 10.3390/molecules191118090

**Published:** 2014-11-05

**Authors:** Yu Wang, Guo-Hua Gong, Wei Zhou, Bin Zhang, Shu-Yin Bao, Cheng-Xi Wei, Jun-Jie Yue, Yan-Fen Zhang

**Affiliations:** 1 Medicinal Chemistry and Pharmacology Institute, Inner Mongolia University for Nationalities, Tongliao 028000, China; 2 State Key Laboratory of Medical Microbiology and Biosafety, Academy of Military Medical Sciences, Biotechnology Institute of Beijing, Beijing 100071, China; 3 Affiliated Hospital of Inner Mongolia University for Nationalities, Tongliao 028000, China; 4 Tongliao Hospital, Tongliao 028000, China

**Keywords:** VirG, apyrase, pull-down

## Abstract

VirG is outer membrane protein of *Shigella* and affects the spread of *Shigella*. Recently it has been reported that apyrase influences the location of VirG, although the underlying mechanism remains poorly understood. The site of interaction between apyrase and VirG is the focus of our research. First we constructed recombinant plasmid pHIS-phoN2 and pS-(v_1–1102_, v_53–758_, v_759–1102,_ v_53–319_, v_320–507_, v_507–758_) by denaturation-renaturation, the phoN2:kan mutant of *Shigella flexneri* 5a M90T by a modified version of the lambda red recombination protocol originally described by Datsenko and Wanner and the complemented strain M90TΔphoN2/pET24a(P_his_phoN2). Second, the recombinant plasmid pHIS-phoN2 and the pS-(v_1–1102_, v_53–758_, v_759–1102,_ v_53–319_, v_320–507_, v_507–758_) were transformed into *E. coli* BL21 (DE3) and induced to express the fusion proteins. Third, the fusion proteins were purified and the interaction of VirG and apyrase was identified by pull-down. Fourth, VirG was divided and the interaction site of apyrase and VirG was determined. Finally, how apyrase affects the function of VirG was analyzed by immunofluorescence. Accordingly, the results provided the data supporting the fact that apyrase combines with the α-domain of VirG to influence the function of VirG.

## 1. Introduction

*Shigella* is a widespread, rapidly growing, Gram-negative bacterium that causes food-borne infections in humans [1]. *Shigella flexneri* can cause bacillary dysentery, bloody diarrhea and the death during childhood [2]. The bacteria are highly virulent, and doses as low as 10 bacteria can infect humans [3]. Every year, about 14,000 cases of shigellosis are reported in the United States. The World Health Organization estimated that at least 163 million cases of bloody diarrhea are caused by *Shigella* spp. in developing countries, with 77% of these cases corresponding to children under 5 years of age, and 1.5 million cases of bloody diarrhea are due to *Shigella* spp. in industrialized countries, with half of the cases corresponding to children younger than 14 years of age [4]. 

The virulence and cell to cell spreading of *Shigella* is dependent on its ability to exploit the actin-based cytoskeleton of host cells to support its motility [5]. The capacity of the bacteria to spread in the cytoplasm and then to move into adjacent epithelial cells is mediated by the bacterial functions encoded by the VirG (IcsA) gene [6]. VirG (IcsA) is a surface-exposed outer membrane protein composed of 1102 amino acids which contains three distinctive domains: the N-terminal signal sequence (residues 1 to 52), the 706-amino acid α-domain (residues 53 to 758), and the 344-amino acid C-terminal β-core (residues 759 to 1102) [7]. The assembly of F-actin near the surface of intracellular *Shigella* is absolutely dependent on the surface presentation of VirG α-domain, and the formation of an actin tail depends on the asymmetric distribution of VirG [8,9].

Apyrase is a periplasmic enzyme which belongs to the family of ATP-hydrolyzing enzymes. It has the ability to hydrolyze nucleoside triphosphates to monophosphates [10], but it lacks the ability to hydrolyze monophosphates [11]. Some research had reported that apyrase was identified by a genetic screen for mutants defective in VirG (IcsA) localization and efficient intercellular spread [12]. Recently, it has been reported that the interaction of apyrase with OmpA might influence VirG exposition and function [13]. Thus, whether it is a direct interaction between apyrase and VirG that affects the function of VirG is not known, so in this paper we study the interaction between apyrase and VirG. 

## 2. Results and Discussion

### 2.1. Apyrase Interacts with VirG

**Figure 1:**
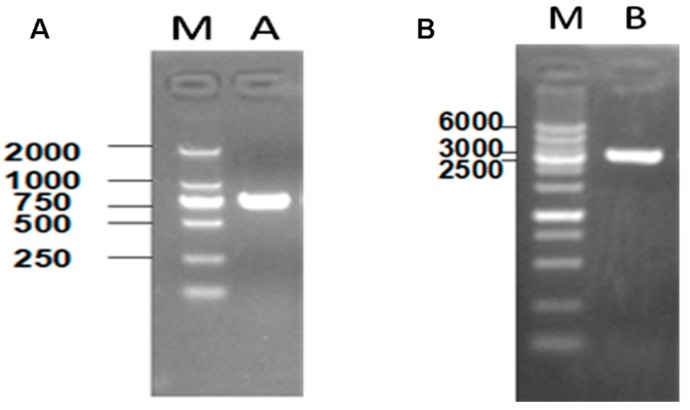
Constructing of recombinant plasmid pET24a (+)-phoN2 and pET32a (+) S-VirG. (**A**) Line A, the phoN2 (741 bp) line. Line M, DL2000 marker; (**B**) Line B, the VirG (3309 bp) line. Line M, DL6000 marker.

We explored the interaction of apyrase and VirG in *Shigella flexneri* 5a M90T. pET24a (+)-phoN2 and pET32a (+) S-VirG were constructed and identified by PCR (Figure 1). The positive clones were sequenced. The result showed the constructed recombinant plasmid pET24a (+)-phoN2 and pET32a (+) S-VirG were not mutants (Tables S1 and S2).

**Figure 2:**
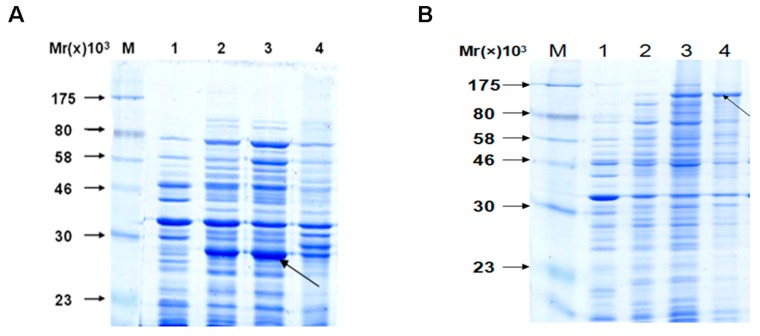
Identification of the fusion proteins apyrase-HIS/VirG-S by SDS-PAGE. (A/B) Line M: molecular weight marker: 175, 80, 58, 46, 30 and 23 kDa; Line 1: whole bacteria before induction; Line 2: whole bacteria after induction; Line 3: supernatant of the ultrasound crushing; Line 4: precipitate of the ultrasound crushing. Notes: (**A**) Analysis expression of apyrase protein; (**B**) Analysis expression of VirG protein.

The fusion protein VirG-S and apyrase-HIS were induced by IPTG. The solubility of fusion protein was analyzed by SDS-PAGE (Figure 2). The fusion proteins were identified in induced bacteria but not in the non-induced. Comparing the content of ultrasound supernatant and ultrasound pellet, most of the fusion proteins were present in supernatant. That means the fusion protein VirG-S and apyrase-HIS were soluble proteins. 

**Figure 3:**
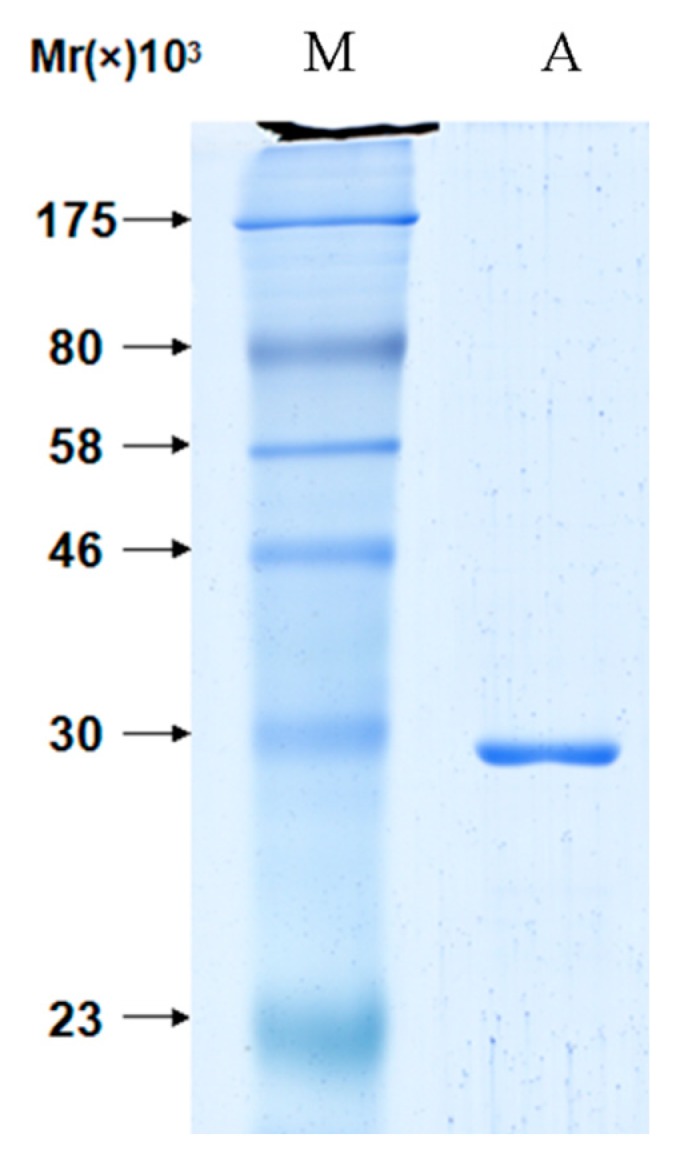
The purified protein apyrase was identified by SDS-PAGE. Line M: molecular weight markers: 175, 80, 58, 46, 30, 23 and 17 kDa; Line A: the purified apyrase protein.

**Figure 4:**
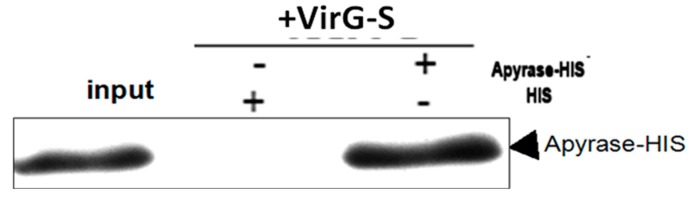
Apyrase protein could bind to VirG protein directly.

The fusion protein VirG-S bound to the S agarose in binding buffer. Aliquots of the agarose containing the covalently attached bait protein were subsequently used for pull-down experiments. To validate the pull-down approach, we analyzed the binding of the candidate interacting protein apyrase after over-expression in *E. coli* BL21 (DE3). The fusion protein apyrase-His was purified by Ni-NTA after induced expression. A single band with a molecular weight of nearly 28 kDa was observed on SDS-PAGE (Figure 3). Then the purified protein apyrase-His was incubated with the S agarose with attached bait protein. After extensive washing, the agarose was collected. The protein-protein interaction can be examined by western blotting (Figure 4). The results showed that VirG-S can precipitate apyrase, while only contain S protein cannot. This showed that there is interaction between apyrase and VirG. It means apyrase affected the location of VirG by direct interaction of the two proteins. Next the site of interaction between apyrase and VirG was identified by pull-down.

In the specific time and space, the specific function of protein is completed by interacting with other proteins to form large complexes, but not by acting independently [14]. The interaction of proteins plays an important role in activating gene transcription of organisms [15], signal transduction [16] and biological [17]. Pull-down technology is an effective method for investigating the interactions of proteins [18,19]. It involves two aspects: (1) it can identify unknown protein interactions with the target protein; (2) it can also identify the interactions between proteins [20].

### 2.2. The Interaction between Apyrase and Residues 507–758 of the α-Domain Was Identified 

**Figure 5:**
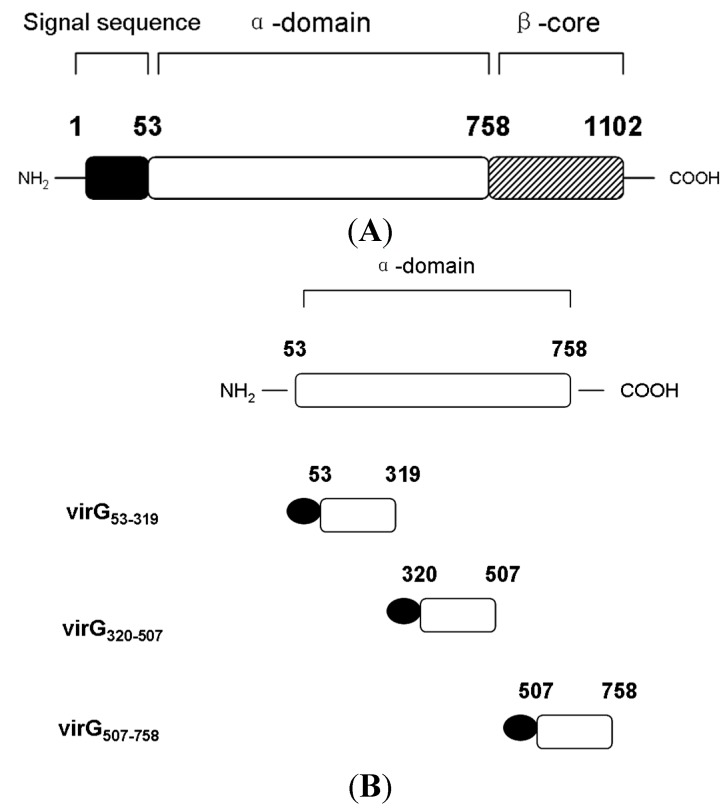
Schematic diagram of the structure of VirG and construction of S-α-domain fusion proteins. (**A**) The structure of VirG contains three domains: N-terminal signal sequence, α-domain and β-core. (**B**) The portions of VirG α-domain fusing the S tag by the pET32a(+)S vector.

VirG is a surface-exposed outer membrane protein composed of 1102 amino acids which contains three distinctive domains: the N-terminal signal sequence (residues 1 to 52), the 706-amino acid α-domain (residues 53 to 758), and the 344-amino acid C-terminal β-core (residues 759 to 1102) [7] (Figure 5A). 

**Figure 6:**
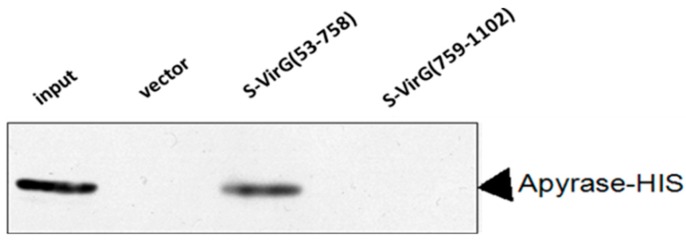
Apyrase protein could bind to the α-domain of VirG protein directly.

**Figure 7:**
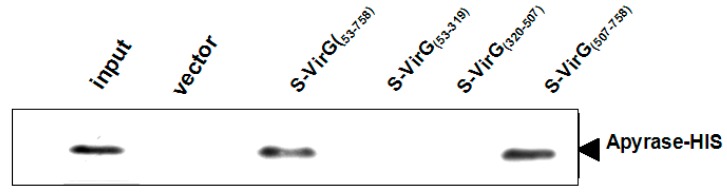
Apyrase protein could bind to 507–758 of α-domain directly.

First we divided the full length VirG into two parts: VirG_(53–758)_, VirG_(759__–1102)_ (the result of induced expression is seen in Figure S1A,B). A pull-down experiment found that apyrase and the α-domain of VirG interacted and the target proteins VirG_(53–758)_-S, VirG_(759__–1102)_-S can be detected after the pull-down experiment (Figure 6 and Figure S2A). Second we divided the α-domain of VirG into three parts (Figure 5B): VirG_53–319_, VirG_320–507_, VirG_507–758_ (the result of induced expression can be seen in Figure S1C–E)_. _ A pull-down experiment found that apyrase and residues 507–758 of the α-domain interacted and the target proteins VirG_53–319_-S, VirG_320–507_-S, VirG_507–758_-S can also be detected after the pull-down experiment (Figure 7 and Figure S2B). The α-domain of VirG is translocated by the membrane pore onto the *Shigella* surface [21], the *N*-terminal 2/3 of the α-domain which includes six Gly-rich repeats, is important for the actin assembly of *Shigella* into a host [22]. The C-terminal 1/3 of the α-domain is essential for the asymmetric distribution of VirG [23]. According to our results, apyrase interacts with residues 507–758 of the α-domain to affected the asymmetric distribution of VirG.

### 2.3. The Function of VirG (IcsA) Was Influenced in ΔphoN2 

**Figure 8:**
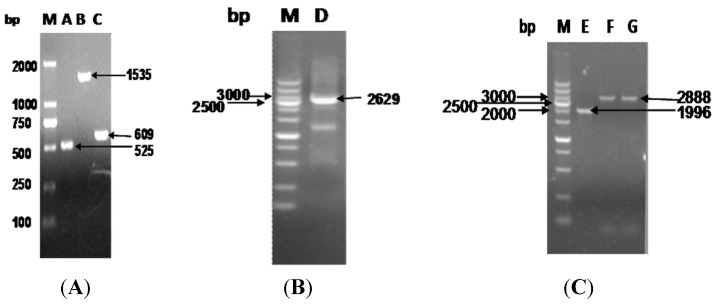
ΔphoN2 was constructed by a modified version of the lambda red recombination protocol. (**A**) Line A: the upstream gene of phoN2 line; Line B: downstream gene of phoN2 line; Line C: the kan gene line; Line M: DL2000 marker. (**B**) Line D: the gene phoN2 homologous recombination fragment line. (**C**) Line E: identification of the M90T wild strain; Line F/G: identification of M90T ΔphoN2:kan.

**Figure 9:**
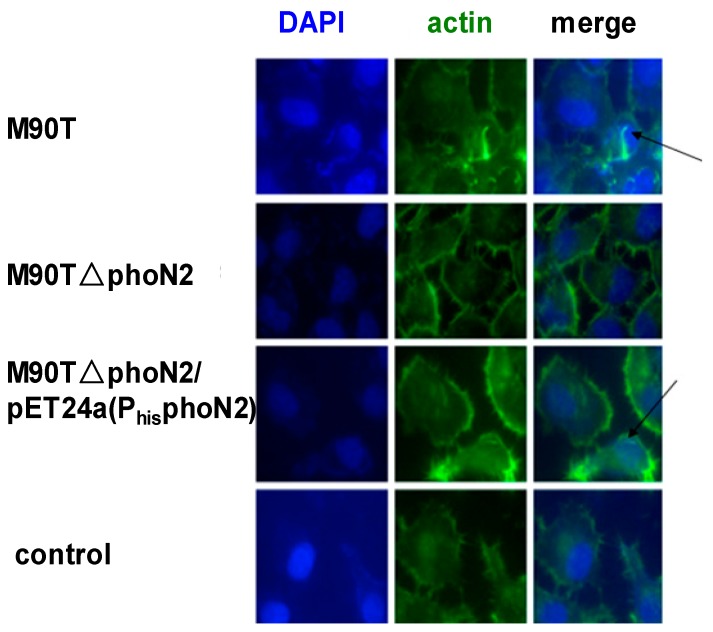
Immunofluorescence assay analysis of actin.

The ΔphoN2 mutations in *S. flexneri* were constructed by a modified version of the lambda red recombination protocol originally described by Datsenko and Wanner [24] (Figure 8). To confirm the effect of apyrase, we carried out an immunofluorescence assay in HeLa cells infected with *Shigella* wild-type, ΔphoN2 and ΔphoN2/pET24a (P_his_phoN2) for 4.5 h. In this method, *Shigella* wild-type infected HeLa cells displayed polar-orientated actin tails (Figure 9). However, the phenomenon of actin polymerization was not detected in ΔphoN2 strain-infected HeLa cells. Located VirG (IcsA) related with actin polymerization has been reported [8]. The reason for the change of actin polymerization in *Shigella* wild-type and ΔphoN2 strains infected HeLa cells involves the altered distribution of VirG on the surface of ΔphoN2 strain and reduces the overall efficiency of spreading of ΔphoN2 strains [12]. Through complement experiments, actin polymerization was observed in ΔphoN2/pET24a (P_his_phoN2) stain-infected HeLa cells. From these results we can see that the protein apyrase may affect *S. flexneri* motility within host cells by VirG in accordance with previous reports [12].

## 3. Experimental Section 

*S. flexneri* 5a M90T was a gift from the Molecular Microbiology Laboratory, MPI für Infektionsbiologie (Berlin, Germany); *E. coli* DH5α and *E. coli* BL21 (DE3) were obtained from TIANGEN (Beijing, China). The vectors pET-24a(+) and pET-32a(+) were obtained from Novagen (Beijing, China); The vector pET-32a(+)S was constructed in our laboratory. PrimeSTAR HS DNA Polymerase, T4 DNA Polymerase and isopropyl β-d-thiogalactoside (IPTG) were purchased from the TaKaRa (Dalian, China).

### 3.1. Construction of Recombinant Plasmids and Mutations in S. flexneri

Genomic DNA was isolated from *S. flexneri* 5a M90T using the Tiangen TIANamp Bacteria DNA kit. The phoN2 gene was amplified from genomic DNA of *S. flexneri* 5a M90T using primers (Table 1). These primers introduced Nde1 and Xho1 restriction sites flanking the sequence. The amplified DNA was sub-cloned into the Nde1/Xho1 sites of vector pET24a (+) to generate a recombinant pHIS-phoN2 plasmid. The construct was verified by sequencing. The plasmid of *S. flexneri* 5a M90T was used for cloning and protein expression. Full length VirG and fragments of VirG (1–1102, 53–758, 759–1102, 53–319, 320–507, 507–758) were amplified. The amplified products were cloned into pET32a (+) S as an N-terminal S-tag fusion. The constructs were verified by sequencing. 

The phoN2:kan mutant of *S. flexneri* 5a M90T was constructed by a modified version of the lambada red recombination protocol originally described by Datsenko and Wanner [24]. We amplified the sequences located directly 500 bp upstream and 500 bp downstream of the phoN2 gene by PCR using the primers A-P1/A-P2 (Table 1) and A-P3/A-P4 (Table 1). The PCR products and the *kan* cassette amplified from the plasmid pKD4 were cloned into pMD-18T to generate the targeting box (upsteam arm-*kan*-downsteam arm) for recombination. *S. flexneri* strain M90T carrying pKD46 containing the red recombinase genes was grown at 30 °C in the presence of 10 mM arabinose (to induce the recombinase genes) and transformed with the gel-purified PCR product of the targeting box amplified using primers A-PF and A-PR (Table 1). Recombinants were selected on LB plates containing kanamycin (50 µg/mL). For genetic complementation, this plasmid pHIS-phoN2 was introduced into strain M90TΔphoN2 to generate the complemented strain M90TΔphoN2 /pET24a (P_his_phoN2).

**Table 1:** The primers used in the method.

Name of Primers	Sequence of Primers
VirG_1_–_1102_	F: 5'-GGATCCATGAATCAAATTCACAAATTTTTTT-3'
	R: 5'-GCTCGAGTCAGAAGGTATATTTCACACCC-3'
VirG_53_–_779_	F: 5'-GGATCCATGACTCCTCTTTCGGGTA-3'
	R: 5'-CTCGAGTCAGCGCCATGTGTGAATA-3'
VirG_758_–_1102_	F: 5'-CGGGATCCCGAGCTAGTTCACA-3'
	R: 5'-CCGCTCGAGTCAGAAGGTATATTTCACA-3'
VirG_53_–_319_	F: 5'-GGATCCATGACTCCTCTTTCGGGTA-3'
	R: 5'-CTCGAGTCATGTTCCATCATCTTCT-3'
VirG_320_–_507_	F: 5'-GGATCCATGCAAAATGTAGCAGGTA-3'
	R: 5'-CCTCGAGTCAACTAACAGTAAGTTCA-3'
VirG_507_–_779_	F: 5'-GGATCCATGAGTACTATTCTGGCAG-3'
	R: 5'-CTCGAGTCAGCGCCATGTGTGAATA-3'
VirG_591_–_758_	F: 5'-CGGGATCCAGTGGGACTGTGCTAAT-3'
	R: 5'-CCGCTCGAGTCAGCGACTACTCATTTGA-3'
phoN2	F: 5'-AATTCCATATGATGAAAACCAAAAAC-3'
	R: 5'-CTCGAGTGGGGTCAGTTCATTG-3'
A-P1	GGTCAAGAGCTTTTTCCCTACT
A-P2	GCCTTCAGAGCATTTGCTGA
A-P3	TAGCGAAGCCAAAAAAGAGT
A-P4	ACTTAGTAAAGATGGTGCCAAC
A-P5	TCAGCAAATGCTCTGAAGGCGTGTAGGCTGGAGCTGCTTC
A-P6	ACTCTTTTTTGGCTTCGCTAATGGGAATTAGCCATGGTCC
A-PF	GCGTATCCTTAACTCTCTGCCTT
A-PR	GGCGATAAAAAAGCTGATATAGC

### 3.2. Protein Expression

The pHIS-apyrase and pS-(V_1–1102_, V_53–758_, V_759–1102,_ V_53–319_, V_320–507_, V_507–758_) vectors were used to transform into *E. coli* BL21 (DE3) and plated on Luria-Bertani (LB) plates containing appropriate antibiotics. LB broth (100 mL), containing antibiotics, was inoculated with 1 mL of an overnight culture and grown at 37 °C until they reached an optical density (OD_600_) of approximately 0.8. Cultures were then cooled on ice to 20 °C and induced with 1 mM of isopropyl β-d galactosidase (IPTG). Cultures were then incubated at 37 °C for 4 h and bacteria were harvested by centrifugation at 6000 r/min for 10 min (4 °C) and washed with ice-cold phosphate buffered saline (PBS). After sonication, a second centrifugation at 13,000 rpm for 15 min (4 °C) was performed to clear the lysate.

### 3.3. Purification of Proteins

The pHIS-apyrase protein was purified using a Ni-NTA Purification System, and insoluble material was removed by centrifugation (12,000 rpm, 15 min, 4 °C). Supernatants were filtered through 0.2 μm sterile Acrodisc syringe filters with Supor^®^ membrane (Pall, New York, NY, USA) onto 100 µL Ni-NTA resin and rotated on a rocking platform at 4 °C for 4 h. After centrifugation (3000 rpm, 5 min, 4 °C), the resin was washed three times in a wash buffer (10 mM Tris-HCl, 10 mM imidazole, 0.5 M NaCl). The pHIS-apyrase was then eluted with increasing concentrations of imidazole (0.5 M).

The pS-(V_1–1102_, V_53–779_, V_759–1102,_ V_53–319_, V_320–507_, V_507–779_, V_591–758_) protein was purified using S agarose. Culture suspensions were thawed on ice and sonicated (as described above), and insoluble material was removed by centrifugation (12,000 rpm, 15 min, 4 °C). Supernatants were filtered through 0.2 μm sterile Acrodisc syringe filters with Supor^®^ membrane (Pall) onto S agarose and rotated on a rocking platform at 4 °C for 4 h. Beads were collected by centrifugation (3000 rpm, 5 min, 4 °C) and washed three times in a wash buffer (20 mM Tris-HCl, 150 mM NaCl, 1% Triton X (v/v)-100). The fusion proteins were stored in S agarose beads at 4 °C. It can be used for pull-down assays.

### 3.4. Pull-Down Assays

In order to investigate protein-protein interactions between apyrase and VirG, pull-down assays were carried out. Briefly, S agarose beads bound to prey protein (S or S-(V_1–1102_, V_53–758_, V_759–1102,_ V_53–319_, V_320–507_, V_507–758_)) were rotated on a rocking platform for 4 h at 4 °C. The agarose beads were then briefly centrifuged (3000 rpm, 5 min, 4 °C), and the supernatant was discarded. The agarose beads were then resuspended and incubated with *E. coli* lysate containing bait protein (pHIS-Apyrase) for 4 h at 4 °C. Beads were collected by brief centrifugation and washed seven times in a wash buffer (20 mM Tris-HCl, 150 mM NaCl, 1% Triton X (v/v)-100). Washed beads were resuspended in 1× SDS-PAGE loading buffer and boiled for 5 min prior to SDS-PAGE separation.

### 3.5. Western Blot Analysis

Samples were separated by SDS-PAGE on a 12.5% gel before transfering to an Immobilon-P PVDF membrane (Bio Rad, Berkeley, CA, USA). The membranes were blocked with 5% (w/v) skim milk in 0.1% Tween 20/TBS (10 mM Tris-HCl, pH 7.6, 150 mM NaCl) and incubated for 1 h at 25 °C. Then primary antibodies in antibody block buffer (5% (w/v) skim milk in 0.1% Tween 20/TBS) and incubated at 4 °C overnight. The following antibodies were used at the indicated dilution and incubated for 1 h at 25 °C. Blots were visualized using the chemiluminescent kit (Santa Cruz, Dallas, TX, USA).

### 3.6. Immunofluorescence Microscopy

Hela cells were grown on 15.6 mm glass-bottom dishes for 24 h and infected with *Shigella* wild-type, ΔphoN2, ΔphoN2/pET24a (P_his_phoN2) and controlled at a multiplicity of infection (moi) of 100. The infected cells were centrifuged (3000 rpm for 1 min at 37 °C) and incubated at 37 °C/5% CO_2_ for 1 h. Then cells were washed three times with PBS and incubated at 37 °C/5% CO_2_ for 3.5 h. After incubation, the infected cells were fixed using a conventional method (4% paraformaldehyde). The fixed cells were used for immunofluorescence. Samples were visualized on a Nikon Ti-u system (Nikon, Osaka, Japan) and images were analyzed. Nuclei were visualized with DAPI staining; actin was stained for F-actin.

## 4. Conclusions 

In conclusion, that apyrase influenced the localization of VirG and intercellular spread has been reported. Our results also proved this viewpoint (Figure 9), but the molecular mechanism of this phenomenon is still not clear. It is significant to understand the interaction of apyrase and VirG. That apyrase combines with the α-domain of VirG has been identified by pull-down. Our results show that apyrase specifically binds to the 507–758 residue site of the VirG α-domain. We are currently using this assay to look for the specific interaction sites of apyrase and VirG. The direct interaction of VirG protein with apyrase to exert its function is proved in our research. It has been reported that hsp70 also can affect the localization of VirG [25]. Whether other proteins would affect the localization of VirG remains an open question. What is the relationship among the three proteins? These problems are the subject of our next research. This paper also provides a theoretical basis for understanding the pathogenesis of *Shigella*.

## Supplementary Materials

Supplementary materials can be accessed at: http://www.mdpi.com/1420-3049/19/11/18090/s1.

## Supplementary Files

Supplementary File 1
