# Supplementary material for: Analysis on the Interaction Domain of VirG and Apyrase by Pull-Down Assay
Source: Molecules. 2014 Nov 5;19(11):18090–101. doi: 10.3390/molecules191118090 (PMC6271496; doi:10.3390/molecules191118090)

Supplementary Materials

Table S1. Sequence analysis of apyrase gene and its encoding amino acids.

ATG AAA ACC AAA AAC TTT CTT CTT TTT TGT ATT GCT ACA AAT ATG ATT TTT ATC CCC TCA
M K T K N F L L F C I A T N M I F I P S
GCA AAT GCT CTG AAG GCA GAA GGT TTT CTC ACT CAA CAA ACT TCA CCA GAC AGT TTG
A N A L K A E G F L T Q Q T S P D S L
TCA ATA CTT CCG CCG CCT CCG GCA GAG GAT TCA GTA GTA TTT CTG GCT GAC AAA GCT CAT
S I L P P P P A E D S V V F L A D K A H
TAT GAA TTC GGC CGC TCG CTC CGG GAT GCT AAT CGT GTA CGT CTC GCT AGC GAA GAT GCA
Y E F G R S L R D A N R V R L A S E D A
TAC TAC GAG AAT TTT GGT CTT GCA TTT TCA GAT GCT TAT GGC ATG GAT ATT TCA AGG GAA
Y Y E N F G L A F S D A Y G M D I S R E
AAT ACC CCA ATC TTA TAT CAG TTG TTA ACA CAA GTA CTA CAG GAT AGC CAT GAT TAC GCC
N T P I L Y Q L L T Q V L Q D S H D Y A
GTG CGT AAC GCC AAA GAA TAT TAT AAA AGA GTT CGT CCA TTC GTT ATT TAT AAA GAC GCA
V R N A K E Y Y K R V R P F V I Y K D A
ACC TGT ACA CCT GAT AAA GAT GAG AAA ATG GCT ATC ACT GGC TCT TAT CCC TCT GGT
T C T P D K D E K M A I T G S Y P S G
CAT GCA TCC TTT GGT TGG GCA GTA GCA CTG ATA CTT GCG GAG ATT AAT CCT CAA CGT AAA
H A S F G W A V A L I L A E I N P Q R K
GCG GAA ATA CTT CGA CGT GGA TAT GAG TTT GGA GAA AGT CGG GTC ATC TGC GGT GCG
A E I L R R G Y E F G E S R V I C G A
CAT TGG CAA AGC GAT GTA GAG GCT GGG CGT TTA ATG GGA GCA TCG GTT GTT GCA GTA
H W Q S D V E A G R L M G A S V V A V
CTT CAT AAT ACA CCT GAA TTT ACC AAA AGC CTT AGC GAA GCC AAA AAA GAG TTT GAA
L H N T P E F T K S L S E A K K E F E
GAA TTA AAT ACT CCT ACC AAT GAA CTG ACC CCA TAA
E L N T P T N E L T P R

Note: The full-length of the sequence was 741 bp and codes for 247 amino acid residues.

Table S2. Sequence analysis of VirG genes and its encoding amino acids.

ATG AAT CAA ATT CAC AAA TTT TTT TGT AAT ATG ACC CAA TGT TCA CAG GGG GGG GCC GGA
M N Q I H K F F C N M T Q C S Q G G A G
GAA TTA CCT ACG GTA AAG GAA AAA ACA TGC AAA TTG TCT TTT TCT CCT TTT GTT GTT GGT
E L P T V K E K T C K L S F S P F V V G
GCA TCC CTG TTG CTC GGG GGG CCA ATA GCT TTT GCT ACT CCT CTT TCG GGT ACT CAA GAA
A S L L L G G P I A F A T P L S G T Q E
CTT CAT TTT TCA GAG GAC AAT TAT GAA AAA TTA TTA ACA CCT GTT GAT GGA CTT TCT CCC
L H F S E D N Y E K L L T P V D G L S P
TTG GGA GCT GGT GAA GAT GGA ATG GAT GCG TGG TAT ATA ACT TCT TCC AAC CCC TCT
L G A G E D G M D A W Y I T S S N P S
CAT GCA TCT AGA ACT AAG CTA CGG ATT AAC TCT GAT ATT ATG ATT AGC GCA GGT CAT GGT
H A S R T K L R I N S D I M I S A G H G
GGT GCT GGT GAT AAT AAT GAT GGT AAT AGT TGT GGC GGT AAT GGT GGT GAC TCT ATT
G A G D N N D G N S C G G N G G D S I
ACC GGA TCT GAC TTG TCT ATA ATC AAT CAA GGC ATG ATT CTT GGT GGT AGC GGC GGT AGC
T G S D L S I I N Q G M I L G G S G G S
GGT GCT GAC CAT AAC GGT GAT GGT GGT GAG GCT GTT ACA GGA GAC AAT CTG TTT ATA ATA

G A D H N G D G G E A V T G D N L F I I
AAT GGA GAA ATT ATT TCA GGT GGA CAT GGT GGC GAT AGT TAT AGT GAT AGT GAT GGG GGG
N G E I I S G G H G G D S Y S D S D G G
AAT GGA GGT GAT GCC GTC ACA GGA GTC AAT CTA CCC ATA ATC AAC AAA GGG ACT ATT TCC
N G G D A V T G V N L P I I N K G T I S
GGT GGT AAT GGA GGT AAC AAT TAT GGT GAG GGT GAT GGC GGT AAT GGA GGT GAT GCC ATC
G G N G G N N Y G E G D G G N G G D A I
ACA GGA AGC AGC CTC TCT GTA ATC AAT AAG GGC ACG TTC GCT GGA GGC AAC GGA GGT GCT
T G S S L S V I N K G T F A G G N G G A
GCT TAC GGT TAT GGT TAT GAT GGC TAC GGT GGT AAT GCT ATC ACA GGA GAT AAC CTG TCT
A Y G Y G Y D G Y G G N A I T G D N L S
GTA ATC AAC AAT GGA GCT ATT TTA GGC GGT AAT GGT GGA CAT TGG GGG GAT GCT ATA AAT
V I N N G A I L G G N G G H W G D A I N
GGT AGC AAT ATG ACC ATT GCT AAT AGC GGA TAT ATA ATT TCA GGT AAA GAA GAT GAT GGA
G S N M T I A N S G Y I I S G K E D D G
ACA CAA AAT GTA GCA GGT AAT GCT ATC CAC ATC ACT GGT GGA AAC AAT TCA TTA ATA CTC
T Q N V A G N A I H I T G G N N S L I L
CAT GAA GGT TCT GTC ATT ACT GGT GAT GTA CAG GTT AAC AAT TCA TCC ATT CTG AAA ATT
H E G S V I T G D V Q V N N S S I L K I
ATC AAC AAT GAT TAC ACT GGG ACC ACA CCA ACT ATT GAA GGT GAT TTA TGT GCT GGT GAT
I N N D Y T G T T P T I E G D L C A G D
TGT ACA ACT GTT TCA CTA TCA GGT AAC AAA TTC ACT GTT TCA GGT GAC GTT TCT TTT GGT
C T T V S L S G N K F T V S G D V S F G
GAG AAC AGT TCT TTA AAT TTA GCT GGA ATC AGT AGT CTG GAA GCT TCT GGA AAT ATG TCA
E N S S L N L A G I S S L E A S G N M S
TTT GGC AAC AAT GTA AAA GTG GAG GCT ATT ATA AAT AAC TGG GCG CAG AAG GAC TAT AAA
F G N N V K V E A I I N N W A Q K D Y K
CTG CTA AGT GCA GAT AAA GGG ATA ACA GGT TTC AGT GTT TCT AAT ATA TCT ATC ATC AAT
L L S A D K G I T G F S V S N I S I I N
CCG TTA CTC ACT ACT GGT GCT ATT GAC TAT ACA AAA AGC TAT ATC AGT GAC CAG AAT AAA
P L L T T G A I D Y T K S Y I S D Q N K
TTG ATC TAC GGT TTG AGC TGG AAT GAT ACA GAT GGC GAC AGT CAT GGA GAG TTC AAT CTG
L I Y G L S W N D T D G D S H G E F N L
AAA GAA AAC GCT GAA CTT ACT GTT AGT ACT ATT CTG GCA GAT AAT CTC AGC CAT CAT AAT
K E N A E L T V S T I L A D N L S H H N
ATA AAT AGC TGG GAC GGA AAA TCC CTA ACA AAA TCA GGG GAG GGA ACT CTC ATT TTG GCG
I N S W D G K S L T K S G E G T L I L A
GAA AAA AAT ACC TAC TCT GGT TTC ACC AAC ATC AAT GCA GGC ATT CTA AAA ATG GGG ACA
E K N T Y S G F T N I N A G I L K M G T
GTT GAA GCT ATG ACA CGT ACC GCT GGT GTT ATT GTT AAT AAA GGT GCT ACC TTG AAT TTT
V E A M T R T A G V I V N K G A T L N F
TCA GGC ATG AAC CAA ACT GTT AAC ACT TTA TTA AAT AGT GGG ACT GTG CTA ATC AAT AAT
S G M N Q T V N T L L N S G T V L I N N
ATT AAT GCC CCT TTT TTG CCT GAC CCC GTC ATT GTC ACA GGT AAC ATG ACT CTG GAG AAA
I N A P F L P D P V I V T G N M T L E K
AAC GGT CAT GTT ATT CTC AAT AAT AGT TCG TCA AAT GTC GGT CAG ACC TAT GTT CAG AAA
N G H V I L N N S S S N V G Q T Y V Q K
GGT AAT TGG CAT GGA AAG GGC GGA ATA TTA TCT TTG GGC GCG GTT CTC GGC AAT GAC AAC
G N W H G K G G I L S L G A V L G N D N
AGT AAA ACT GAC CGG CTG GAA ATT GCA GGC CAT GCG TCT GGT ATT ACC TAT GTT GCA GTG

S K T D R L E I A G H A S G I T Y V A V

ACA AAT GAG GGA GGC TCT GGA GAT AAA ACT CTT GAA GGT GTT CAA ATT ATT TCG ACA GAT

T N E G G S G D K T L E G V Q I I S T D

TCT TCT GAT AAG AAT GCT TTT ATT CAG AAA GGC CGT ATT GTT GCT GGT AGT TAT GAC TAT

S S D K N A F I Q K G R I V A G S Y D Y

CGC CTG AAA CAG GGC ACT GCA TCT GGA CTG AAT ACC AAT AAG TGG TAT CTA ACT AGT CAG

R L K Q G T A S G L N T N K W Y L T S Q

ATG GAT AAT CAA GAA TCA AAA CAG ATG AGC AAT CAA GAG TCT ACT CAA ATG AGT AGT

M D N Q E S K Q M S N Q E S T Q M S S

CGC CGA GCT AGT TCA CAG CTT GTA TCT TCA CTT AAT TTG GGT GAA GGT AGT ATT CAC ACA

R R A S S Q L V S S L N L G E G S I H T

TGG CGC CCT GAA GCT GGC AGT TAT ATT GCT AAC CTG ATA GCA ATG AAC ACG ATG TTT AGT

W R P E A G S Y I A N L I A M N T M F S

CCT TCT CTC TAT GAC CGA CAC GGT AGC ACT ATT GTT GAT CCT ACT ACA GGT CAG CTC AGC

P S L Y D R H G S T I V D P T T G Q L S

GAA ACC ACC ATG TGG ATT CGT ACT GTT GGT GGA CAT AAT GAG CAT AAT TTA GCT GAT AGA

E T T M W I R T V G G H N E H N L A D R

CAA TTA AAA ACC ACA GCT AAC AGG ATG GTT TAT CAG ATT GGT GGA GAT ATT TTG AAG ACA

Q L K T T A N R M V Y Q I G G D I L K T

AAC TTC ACT GAT CAT GAT GGC TTG CAT GTG GGT ATT ATG GGA GCT TAT GGA TAT CAG GAT

N F T D H D G L H V G I M G A Y G Y Q D

AGC AAA ACT CAT AAT AAG TAT ACT AGT TAT AGT TCA CGA GGA ACT GTG AGC GGT TAT ACT

S K T H N K Y T S Y S S R G T V S G Y T

GCC GGT TTG TAC AGT TCT TGG TTT CAG GAT GAA AAA GAA CGA ACA GGT CTA TAT ATG GAT

A G L Y S S W F Q D E K E R T G L Y M D

GCT TGG TTG CAG TAC AGT TGG TTT AAT AAT ACA GTC AAA GGA GAT GGG TTA ACT GGT GAG

A W L Q Y S W F N N T V K G D G L T G E

AAA TAT TCC AGC AAA GGA ATA ACA GGA GCT TTG GAA GCT GGC TAT ATC TAC CCA ACC ATA

K Y S S K G I T G A L E A G Y I Y P T I

CGC TGG ACT GCT CAT AAT AAT ATT GAC AAC GCA TTG TAT CTC AAT CCA CAA GTC CAG ATA

R W T A H N N I D N A L Y L N P Q V Q I

ACT AGG CAT GGG GTA AAA GCA AAC GAC TAT ATT GAA CAC AAT GGC ACT ATG GTC ACA TCC

T R H G V K A N D Y I E H N G T M V T S

TCT GGG GGC AAT AAT ATT CAA GCA AAA TTG GGA TTG CGT ACA TCC TTA ATT AGT CAG AGT

S G G N N I Q A K L G L R T S L I S Q S

TGT ATC GAT AAG GAG ACT CTT CGT AAG TTC GAA CCA TTT TTG GAA GTG AAT TGG AAA TGG

C I D K E T L R K F E P F L E V N W K W

AGC TCA AAG CAA TAT GGT GTA ATT ATG AAT GGC ATG TCA AAT CAC CAG ATA GGC AAC CGT

S S K Q Y G V I M N G M S N H Q I G N R

AAT GTG ATT GAA CTC AAA ACT GGT GTG GGG GGG CGT CTT GCA GAT AAC CTA AGC ATC TGG

N V I E L K T G V G G R L A D N L S I W

GGA AAC GTA TCT CAG CAA TTG GGT AAT AAC AGT TAC AGA GAC ACC CAA GGT ATT TTG GGT

G N V S Q Q L G N N S Y R D T Q G I L G

GTG AAA TAT ACC TTC TGA

V K Y T F

Note: The full-length of the sequence was 3309 bp and codes for 1102 amino acid residues.

Figure S1. Identification of the fusion proteins VirG$_{(53–758)}$-S, VirG$_{(759–1102)}$-S, VirG$_{(53–319)}$-S, VirG$_{(320–507)}$-S, VirG$_{(507–758)}$-S by western blotting. Line 1: whole bacteria after induction; Line 2: supernatant of the ultrasound crushing; Line 3: precipitate of the ultrasound crushing. (**A**) Analysis the expression of VirG$_{(53–758)}$-S; (**B**) Analysis the expression of VirG$_{(759-1102)}$-S; (**C**) Analysis the expression of VirG$_{(53–319)}$-S; (**D**) Analysis the expression of VirG$_{(320-507)}$-S; (**E**) Analysis the expression of VirG$_{(320–758)}$-S.

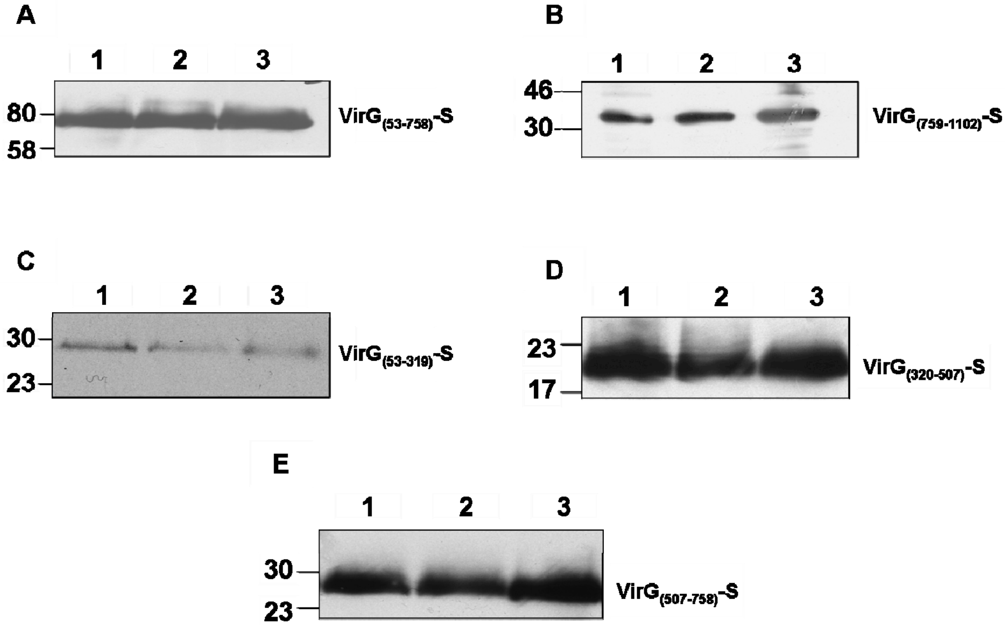

Figure S2. Analysis of the purified proteins VirG$_{(53-758)}$-S, VirG$_{(759-1102)}$-S, VirG$_{(53-319)}$-S, VirG$_{(320-507)}$-S, VirG$_{(507-758)}$-S by western blotting after the pull-down experiment. (**A**) Identification of VirG$_{(53-758)}$-S and VirG$_{(759-1102)}$-S; (**B**) Identification of VirG$_{(53-319)}$-S, VirG$_{(320-507)}$-S and VirG$_{(507-758)}$-S.

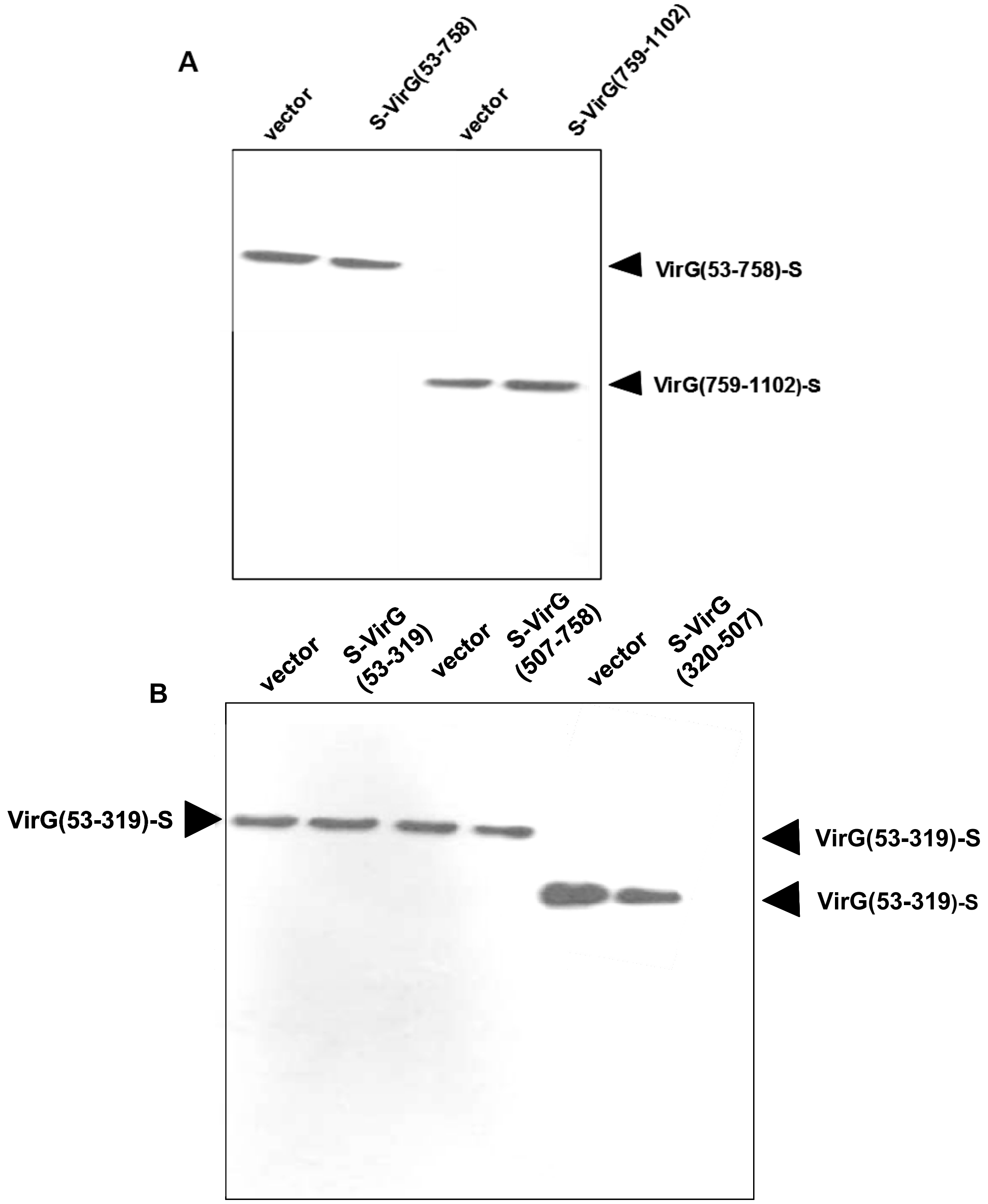

Supplement: Supplementary File 1 [file molecules-19-18090-s001.pdf]
